# Correlation between immunity from BCG and the morbidity and mortality of COVID-19

**DOI:** 10.1186/s40794-020-00117-z

**Published:** 2020-08-28

**Authors:** Dakshitha Wickramasinghe, Nilanka Wickramasinghe, Sohan Anjana Kamburugamuwa, Carukshi Arambepola, Dharmabandhu N. Samarasekera

**Affiliations:** 1 Department of Surgery, Faculty of Medicine, University of Colombo, Colombo, Sri Lanka; 2 Department of Physiology, Faculty of Medicine, University of Colombo, Colombo, Sri Lanka; 3 Department of Community Medicine, Faculty of Medicine, University of Colombo, Colombo, Sri Lanka

**Keywords:** COVID-19, BCG vaccination, Tuberculosis, Fatality

## Abstract

**Background:**

To investigate the association between parameters indicating immunity from BCG at country level (presence of BCG vaccination policy, BCG coverage, age-specific incidence of tuberculosis (TB)) and the morbidity and mortality of COVID-19.

**Methods:**

Country-specific data for COVID-19 cases and deaths, demographic details, BCG coverage and policy, age-specific TB incidence and income level were obtained. The crude COVID-19 cases and deaths per 100,000 population were calculated and assessed against the parameters indicating immunity from BCG using linear regression analysis.

**Results:**

Univariate analysis identified higher income level of a country to be significantly associated with COVID-19 cases (*p* < 0.0001) and deaths (*p* < 0.0001) but not with its case fatality rate. The association between COVID-19 and TB was strongest for TB incidence in patients > 65-years (Cases (r_s_ = − 0.785,*p* = 0.0001)) and deaths (r_s_ = − 0.647,*p* = 0.0001).

Multivariate analysis identified the higher income level of a country and not having a universal BCG vaccination policy to affect the COVID-19 cases. The deaths were inversely affected by the presence of BCG vaccination policy and coverage; and positively by the TB incidence in patients > 65-years.

**Conclusion:**

Significant inverse correlations observed between cases and deaths of COVID-19 and BCG related parameters highlights immunity from BCG as a likely explanation for the variation in COVID-19 across countries.

## Introduction

The COVID-19 outbreak started in December 2019 in Wuhan Province, China [1] and has by now spread to six continents. It is caused by SARS-CoV-2 [2]. It has overwhelmed the health system capacity in many countries. No specific treatment has been effective to date, while the thrust in controlling it is placed on public health interventions. Available literature suggests the mortality to be higher in patients of advanced age and comorbidities [1].

Bacillus Calmette–Guérin (BCG) is a live-attenuated vaccine primarily developed to protect against childhood meningitis and disseminated tuberculosis (TB) [3]. The World Health Organization (WHO) recommends BCG vaccination at birth in countries with both universal and selective vaccination policies [4]. Furthermore, multiple meta-analyses have confirmed the effectiveness of BCG against pulmonary and extra-pulmonary TB in adults [5–7]. It is however assumed that prior exposure to environmental mycobacteria would confer a partial resistance and thereby diminish the response to BCG vaccine. Thus, vaccination in older age groups is only recommended in a few selected scenarios, while there is no universal policy for BCG vaccination of immunocompromised or other high-risk individuals.

Several researchers have suggested an inverse relationship between immunity from BCG and the case and death rates from COVID-19 [8, 9]. Considering the high morbidity and mortality of COVID-19 in the elderly, a possible protective effect through BCG in this group would invariably be of clinical significance. Nevertheless, ecological studies conducted so far have failed to establish this relationship [10]. It could be that these studies have all used a single indicator to depict immunity from BCG, which is commonly the population coverage of BCG vaccination. This value does not reflect the immunisation status of different age groups. At the same time, the BCG immunisation status is also not routinely assessed in different age groups. Therefore, considering the protection that BCG offers against pulmonary and extra-pulmonary TB, we hypothesised that the incidence of TB in different age groups could be used as a surrogate marker to indicate poor immunity from BCG in those age groups.

BCG vaccination is believed to confer a non-specific increase in immunity [11], and acts via both innate and adaptive immune responses. The use of BCG as an immunotherapy in bladder cancer and melanoma [12] is an extension of this theory. We believe this increase in immunity may contribute to the differences in COVID-19 morbidity and mortality.

We further hypothesized that the income level of each country is likely to confound this relationship, as the morbidity and mortality from COVID-19 have been consistently higher in countries with a higher mean GDP. The majority of these countries had also abandoned universal BCG vaccination in the preceding decades.

Thus, the primary aim of this study was to investigate the population level associations of BCG coverage, incidence of TB in different age groups and income status of the countries with the morbidity and mortality of COVID-19.

## Methods

### Data sources

Country-specific data for COVID-19 cases and deaths were retrieved from the Johns Hopkins Coronavirus Resource Centre [13]. Data on population were obtained from the World Bank population data [14]. This included the total population, population of 15–64 years and > 65 years. BCG coverage data were obtained from the BCG Atlas [15] and the World Health Organization data on immunisation coverage [16]. Data on TB burden was obtained from the World Health Organization Global Health Observatory Data Repository [17]. The income level of the country was obtained from the World Bank population data and open data repository [18]. Data were retrieved on 11/04/2020.

To reduce the bias of the availability of testing, we calculated the crude COVID-19 mortality per 100,000 population using population data published by the World Bank.

### Statistical analysis

Data were analysed using IBM SPSS statistics for Windows, version 26.0. Correlations were assessed using the Spearman correlation coefficient (r_s_). Tests applied to assess the significance between groups were Mann-Whitney test for scaled data, Chi-Square test for categorical data, and Kruskal-Wallis test for multiple groups of continuous data.

Linear regression analysis using backward LR method was performed to assess the relationship between COVID-19 and immunity from BCG adjusted for confounders. In the models, the morbidity or mortality were used as the dependent variable, while the independent variables were proxy indicators of the status of immunity from BCG of populations, namely, the presence or absence of a universal BCG vaccination policy, the WHO estimates of the BCG coverage of the total population, the BCG coverage in 1-year olds, TB incidence in 15–64 year age group and TB incidence in > 65-year age group. The income level of each country was also added to the model, as it could confound a true association between the dependent and independent variables. A probability value of < 0.05 was considered statistically significant.

## Results

A total of 225 countries were eligible to be included in the analysis. Tuberculosis and population-level data were available for 214 countries. COVID-19 and BCG coverage data were available for 180 and 175 countries, respectively.

### Distribution of COVID-19 cases and deaths by income level of countries

**Fig. 1:**
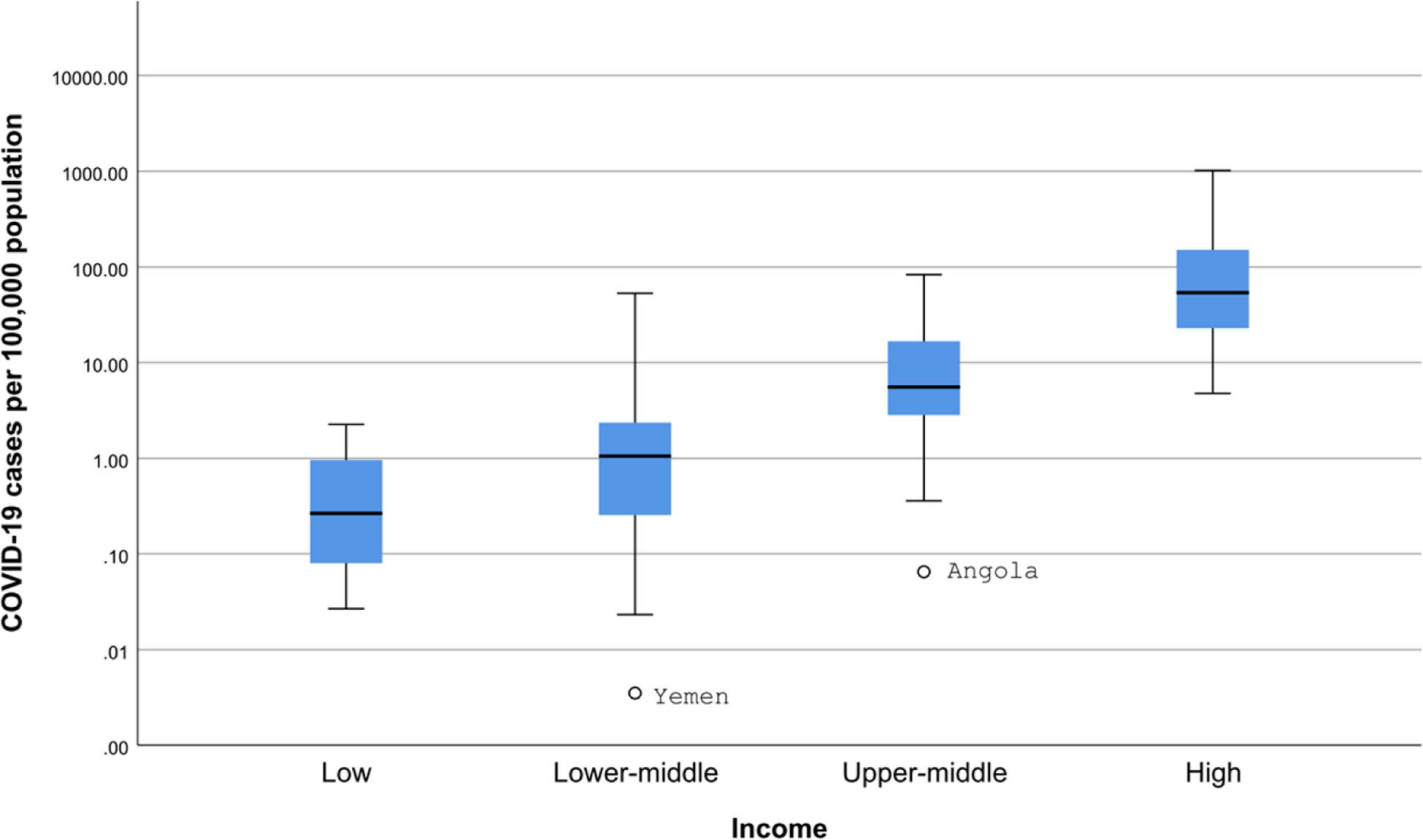
COVID-19 cases per 100,000 population according to the income level of countries. o - 1.5-3 box lengths from the median. * - ≥3 box lengths from the median

**Fig. 2:**
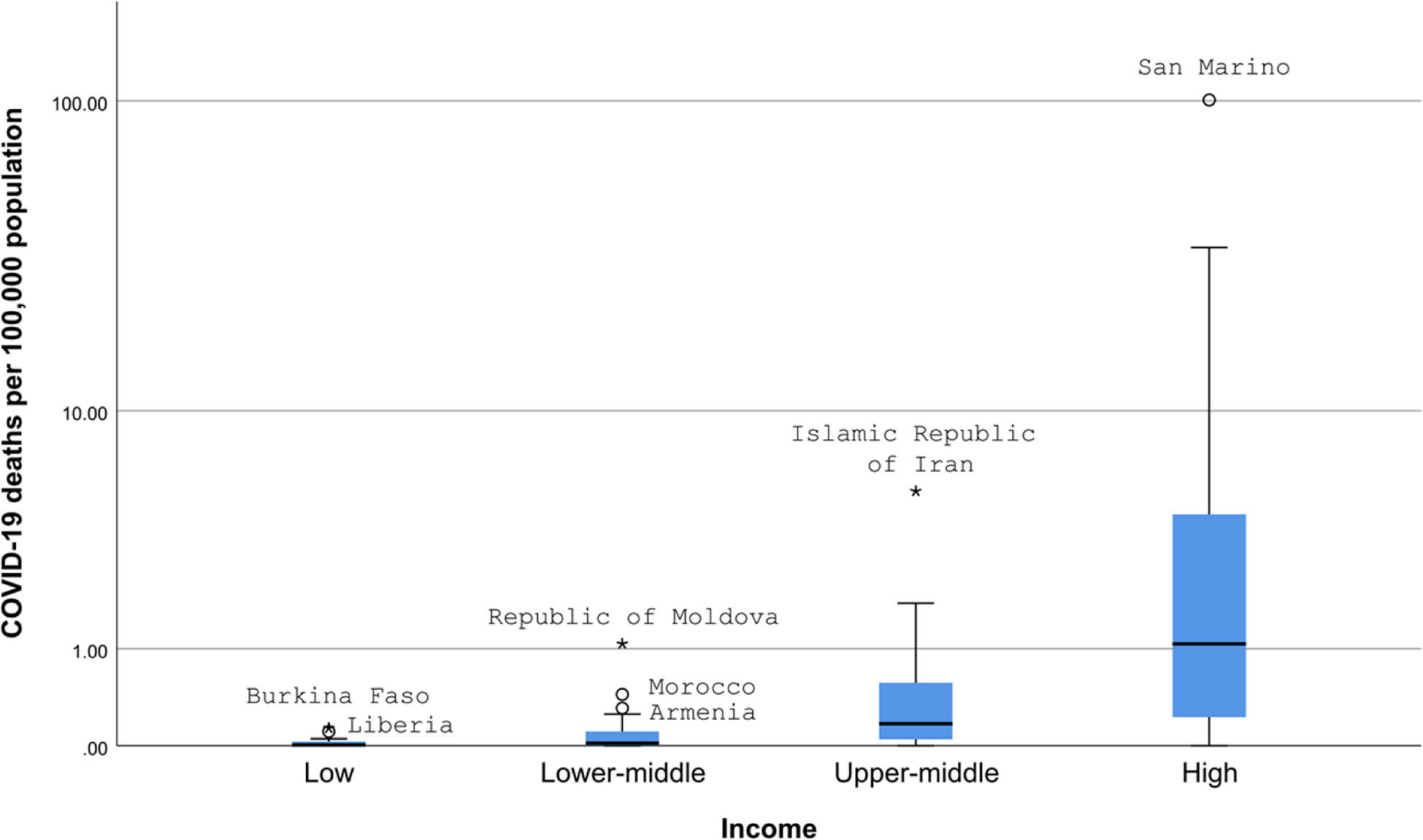
COVID-19 deaths per 100,000 population according to the income level of countries. o - 1.5-3 box lengths from the median. * - ≥3 box lengths from the median

**Fig. 3:**
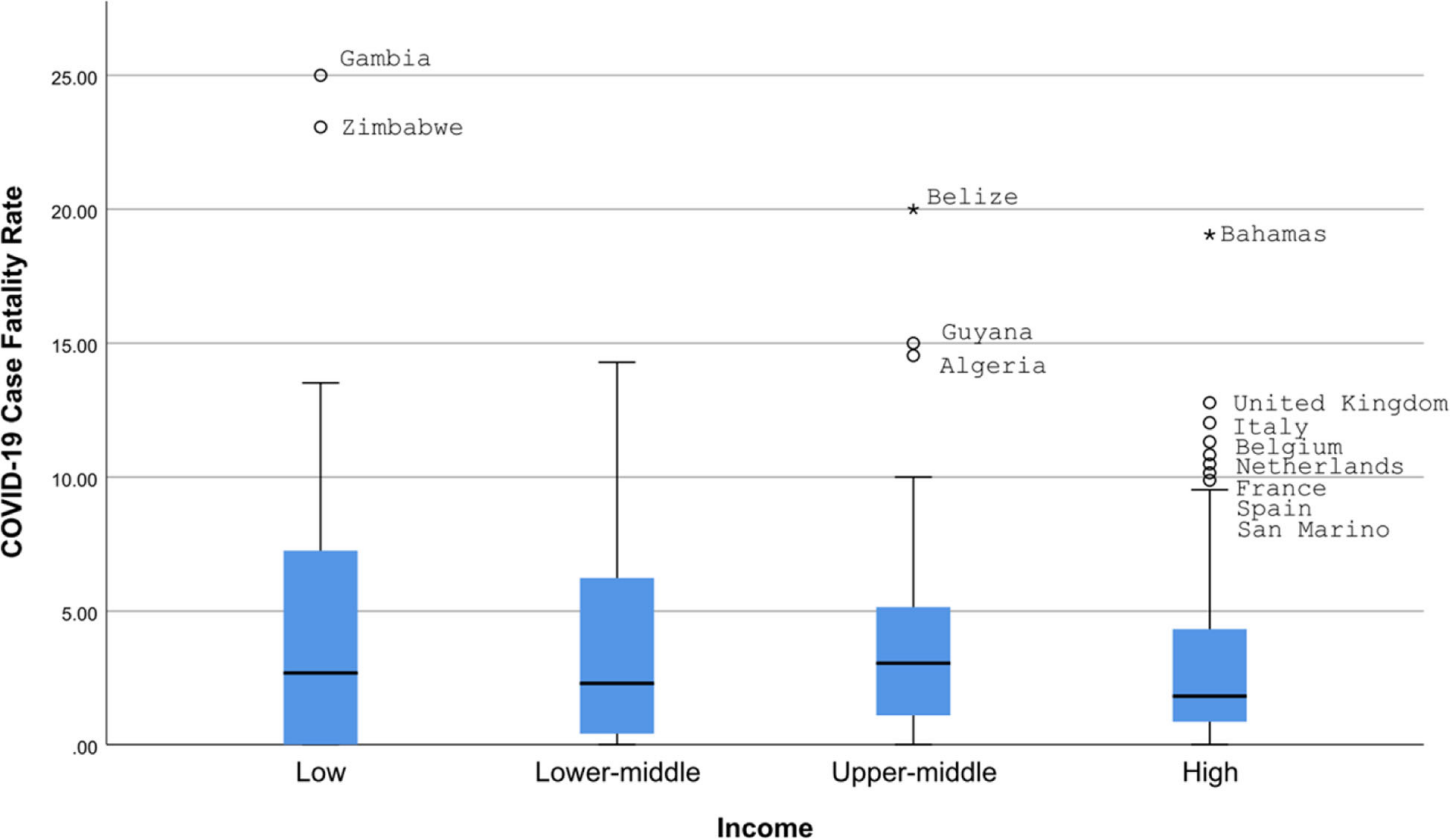
COVID-19 Case Fatality Rate according by income level of the country. o - 1.5-3 box lengths from the median. * - ≥3 box lengths from the median

The COVID-19 cases were significantly different between countries of different income levels (Kruskal-Wallis test, *p* < 0.0001) (Fig. 1). All differences except that between low and lower-middle-income countries were statistically significant (Mann-Whitney test, *p* < 0.0005 for all). Similarly, COVID-19 deaths were significantly different between countries in different income levels (Kruskal-Wallis test, *p* < 0.0001) (Fig. 2). All differences except that between low and lower-middle-income countries were statistically significant (Mann-Whitney test, *p* < 0.005 for all). There was no statistically significant difference in the case fatality rate and the level of income (Fig. 3).

### Relationship of COVID-19 cases and deaths with immunity from BCG at country level

The BCG coverage was inversely correlated with the incidence of TB (r_s_ = − 0.164, *p* = 0.03). However, it showed no significant correlation with the cases or deaths per 100,000 from COVID-19 (*p* = 0.43 and 0.247, respectively). Instead, as shown in Table 1, the countrywide TB incidence had a strong inverse correlation with the COVID-19 cases per 100,000 population (r_s_ = − 0.743, *p* = 0.0001) and COVID-19 deaths per 100,000 population (r_s_ = − 0.611, *p* = 0.0001). Similar correlations were observed for both pulmonary (both bacteriologically confirmed and clinically confirmed) and extra-pulmonary TB. The association between COVID-19 and TB was strongest when comparing the TB incidence in patients over 65-years, in which both COVID-19 cases per 100,000 population (r_s_ = − 0.785, *p* = 0.0001) and COVID-19 deaths per 100,000 population (r_s_ = − 0.647, *p* = 0.0001) showed significant inverse correlations. The correlation between the younger age group (15–64 years) was also significant, but less strong (Cases (r_s_ = − 0.742, *p* = 0.0001) and deaths (r_s_ = − 0.621, *p* = 0.0001)).

**Table 1:** The correlations of COVID-19 cases and death with BCG coverage and incidence of TB

	COVID-19 cases per 100,000 population			COVID-19 deaths per 100,000 population		
Cases	Total population	BCG coverage < 95%	BCG coverage ≥ 95%	Total population	BCG coverage < 95%	BCG coverage ≥ 95%
BCG coverage	0.136 (*p* = 0.091)	0–.064 (*p* = 0.588)	0.176 (*p* = 0.111)	0.061 (*p* = 0.447)	−0.057 (*p* = 0.629)	0.227 (*p* = 0.038)
Pulmonary TB – Bacteriologically confirmed	−0.687 (*p* < 0.0005)	−0.556 (*p* < 0.0005)	− 0.591 (*p* < 0.0005)	−0.570 (*p* < 0.0005)	− 0.574 (*p* < 0.0005)	−0.333 (*p* = 0.002)
Pulmonary TB – Clinically confirmed	−0.699 (*p* < 0.0005)	−0.674 (*p* < 0.0005)	− 0.567 (*p* < 0.0005)	−0.516 (*p* < 0.0005)	− 0.639 (*p* < 0.0005)	−0.246 (*p* = 0.0026)
Extra-pulmonary TB	−0.652 (*p* < 0.0005)	−0.626 (*p* < 0.0005)	− 0.537 (*p* < 0.0005)	−0.541 (*p* < 0.0005)	− 0.607 (*p* < 0.0005)	−0.314 (*p* = 0.004)
Overall TB incidence	−0.743 (*p* < 0.0005)	−0.644 (*p* < 0.0005)	− 0.663 (*p* < 0.0005)	−0.611 (*p* < 0.0005)	− 0.645 (*p* < 0.0005)	−0.379 (*p* < 0.0005)
TB incidence in 15–64-year group	−0.742 (*p* < 0.0005)	− 0.649 (*p* < 0.0005)	−0.677 (*p* < 0.0005)	− 0.621 (*p* < 0.0005)	−0.646 (*p* < 0.0005)	− 0.405 (*p* < 0.0005)
TB incidence in > 65-year group	− 0.785 (*p* < 0.0005)	− 0.701 (*p* < 0.0005)	−0.697 (*p* < 0.0005)	− 0.647 (*p* < 0.0005)	−0.704 (*p* < 0.0005)	− 0.382 (*p* < 0.0005)

*BCG* Bacillus Calmette–Guérin vaccine

*TB* Tuberculosis

We further assessed the relationship between the incidence of TB and COVID-19 cases and deaths stratified by countries with BCG coverage < 95% vs. ≥95% (Table 1). All correlations with COVID-19 deaths were higher in countries with BCG coverage < 95% than ≥95%. Similar observations were observed for COVID-19 cases except for three parameters, which were marginally higher in countries with higher BCG coverage. The Case Fatality Rate (CFR) showed a weak inverse correlation with TB deaths in countries (r_s_ = − 0.208, *p* = 0.006).

**Fig. 4:**
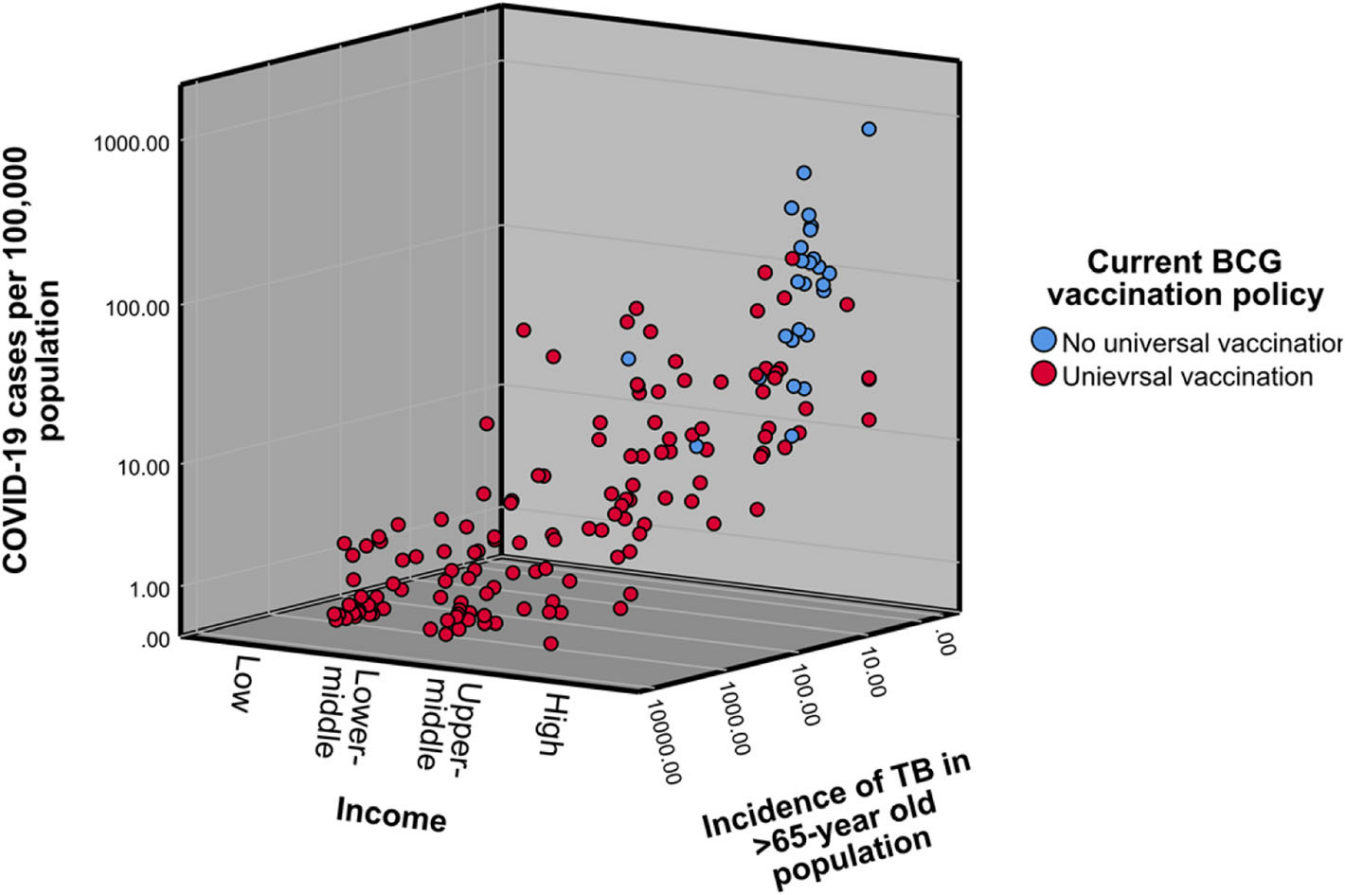
COVID-19 Cases per 100,000 population according to the income status and incidence of TB in the population over 65-years

**Fig. 5:**
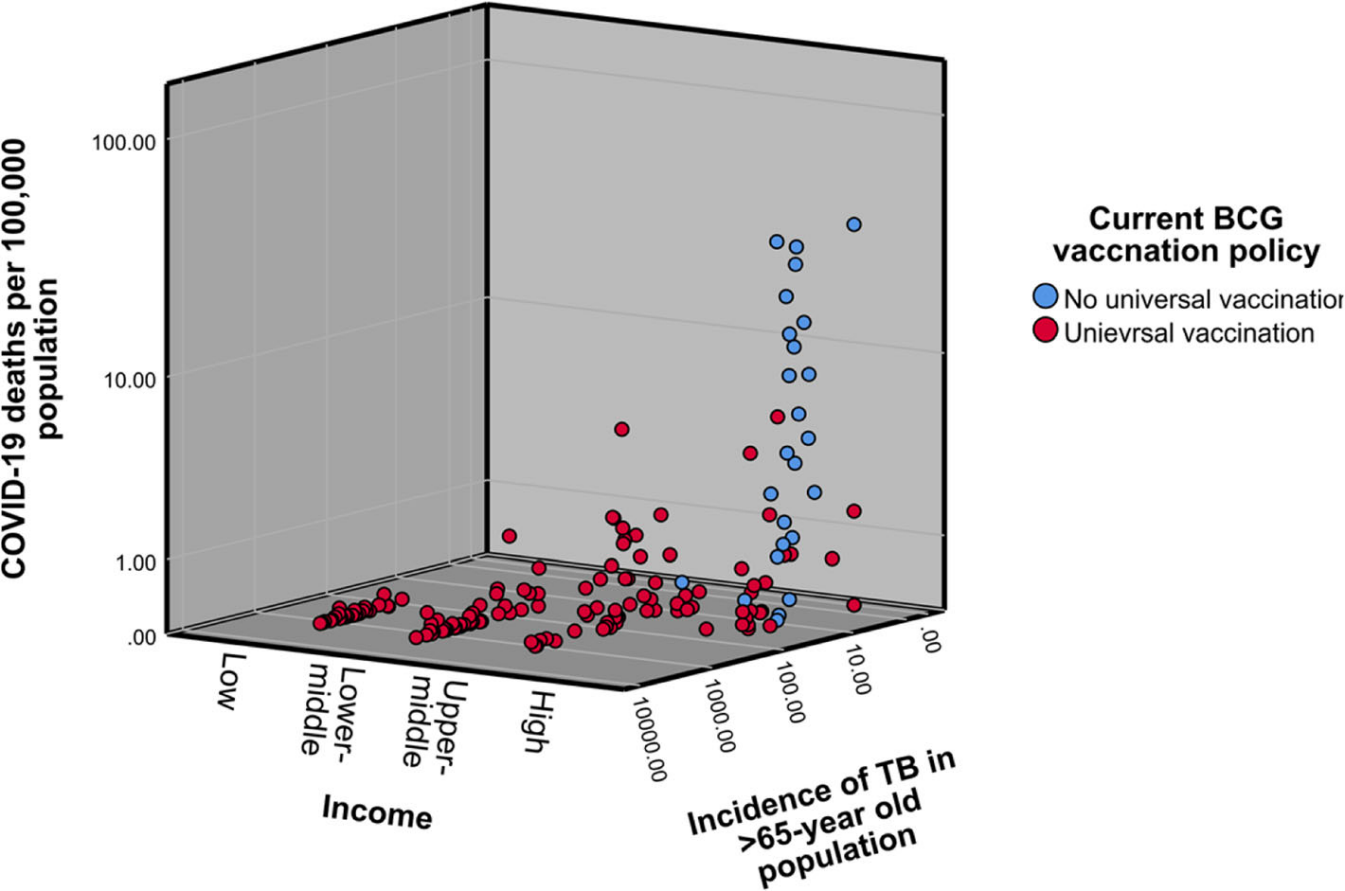
COVID-19 deaths per 100,000 population according to the income status and incidence of TB in the population over 65-years

When stratified according to income level, the BCG coverage showed a statistically significant moderate inverse correlation with both cases (r_s_ = − 0.545, *p* < 0.0005) and deaths (r_s_ = − 0.564, *p* < 0.0005) within the high-income group countries. No other income group showed a statistically significant correlation with BCG coverage. The correlation of COVID-19 cases and deaths with the incidence of TB in the population over 65-years progressively increased according to the income status of the country (Figs. 4 & 5).

### Multivariate analysis

Regarding the linear regression performed for COVID-19 cases per 100,000 population, the model was statistically significant (F (3,62) = 27.028, *p* < 0.0005) and explained 54.6% of the variance observed. It identified a statistically significant effect on the caseload from the income status of a country; and a significant inverse effect from the presence of BCG vaccination policy. The WHO estimates of the BCG coverage of the population were not statistically significant (Table 2).

**Table 2:** Linear regression analysis for COVID-19 cases per 100,000 population

Covariate	Regression coefficient	Significance
(Constant)	68.647	0.001
WHO estimates of BCG coverage	−0.331	0.085
Income	11.358	0.001
Current BCG policy status	−58.300	0.000

*WHO* World Health Organization

*BCG* Bacillus Calmette–Guérin vaccine

With regard to the linear regression performed for COVID-19 deaths per 100,000 population, the model was statistically significant (F(3,144) = 43.694, *p* < 0.0005) and explained 46.6% of the variance observed. It identified a statistically significant effect on deaths, from the incidence of TB in > 65-year old population, and an inverse effect from both the presence of BCG vaccination policy and WHO estimates of the BCG coverage of the population (Table 3).

**Table 3:** Linear regression analysis for COVID-19 deaths per 100,000 population

Covariate	Regression coefficient	Significance
(Constant)	8.633	0.000
TB incidence in 65 plus per 100,000	0.001	0.017
Current BCG policy status	−3.100	0.000
WHO estimates of BCG coverage	−0.054	0.000

*TB* Tuberculosis

*BCG* Bacillus Calmette–Guérin vaccine

*WHO* World Health Organization

The linear regression analysis using the case fatality rate as the dependent variable did not provide a statistically significant result.

## Discussion

This study examines a previously observed association between the proxy measures of immunity from BCG of countries and the morbidity and mortality from COVID-19. We explored this association further by adopting the TB incidence in different age-groups as a surrogate of BCG coverage, as no age-specific BCG coverage data are currently available.

In summary, our findings indicate that the BCG coverage did not have a significant correlation with the cases or deaths from COVID-19. The countrywide TB incidence as well as pulmonary and extra-pulmonary TB incidence had strong inverse correlations with both cases and deaths. This association was strongest when comparing the TB incidence in patients over 65-years. Furthermore, all correlations with COVID-19 deaths were higher in countries with BCG coverage < 95% than ≥95%. Our multivariate analysis identified the presence of a universal BCG vaccination policy to inversely affect both cases and deaths from COVID-19.

The impact of BCG vaccination on the transmission of *Mycobacterium tuberculosis* is limited, as it does not prevent the reactivation of latent pulmonary infection, which is the principal source of bacillary spread in the community. There is significant variation in the effectiveness of BCG vaccination, and prior exposure to environmental mycobacteria is thought to influence this [19]. This variation may also explain the inconclusive evidence shown for the protection of BCG vaccination against COVID-19. In contrast, a reduced incidence of TB is a more direct indicator of the efficacy of BCG vaccination. This may explain our observations.

The presence of a universal BCG vaccination policy showed a stronger correlation than the BCG coverage, for both cases and deaths from COVID-19. In concurrence, in the study by Sala et al. [9], BCG coverage only accounted for 12.5% of the variance observed. The results of Miller et al. [20] suggest that factors increasing the fraction of the elderly population being vaccinated against BCG, like an earlier year of initiation of a universal BCG vaccination policy, had a good correlation with COVID-19 cases and deaths.

BCG vaccination is believed to confer a non-specific increase in immunity [11]. It acts via both innate and adaptive immune responses, and in the latter, via both T and B cells. This theory is applied when BCG is used as an immunotherapy in the management of melanoma and bladder cancer [12]. BCG induces long-lived memory B-cells [21]. BCG also induces and maintains tissue-resident CD4 T cells in the lungs [22]. COVID-19 is presumed to enter the body through the lungs [21]. These tissue-resident CD4 cells may therefore influence the entry into the body and therefore contribute to lower cases and deaths in countries with BCG vaccination. A clinical trial has already begun testing the utility of BCG for boosting immunity against COVID-19 [23].

Over 90% of the TB cases in the elderly are due to reactivation of primary infection [24]. Rarely, previously infected older persons who have eliminated the viable tubercle bacilli may revert to a “naïve” immunologic status and are at risk of reinfection [25]. These mechanisms may imply the loss of the initial protection from BCG vaccination. The better correlation of COVID-19 cases and deaths with the TB incidence in older ages groups than the younger groups also supports this. The changes occurring in the immune system with ageing may also contribute to this [26, 27]. The reduced incidence of TB could also represent an overall improvement in preventive care. The results we observed may be related to an unknown organism or antigen closely correlated to both TB and COVID-19, similar to the hygiene hypothesis [28].

The mortality from COVID-19 is reportedly higher in patients who are older and comorbid [1, 29]. In the study by Zhou et al. [1], the interquartile range (IQR) of the age of survivors and non-survivors was 45–58 and 63–76 years, respectively. This shows a clear demarcation with possibly no-overlap. Our analysis only identified the TB incidence in > 65-year olds to be significantly inversely correlated with deaths. The incidence in the total population and the 15–64-year group showed smaller correlations. We used the incidence of TB is a surrogate marker of the immunity offered by the BCG vaccination. Lesser deaths observed in populations where there is a lesser number of TB in the > 65-year olds support this hypothesis. There is no worldwide age-specific case and mortality data for COVID-19 and we were therefore unable to investigate this hypothesis further.

The relationship between the income and COVID-19 cases is paradoxical to observations during previous epidemics [30, 31]. High-income countries generally depend on larger budgetary allocations for secondary preventive strategies to contain an epidemic within the health system capacity. Their outcomes during the COVID-19 pandemic have remained worse than countries with lower income due to a multitude of reasons. The income level remained independently associated with the case number in our multivariate analysis and has been reported by other researchers as well [3, 8, 20, 32]. In the subgroup analysis, we observed that, for high-income countries, the correlation between cases and deaths and the degree of BCG coverage was higher. Most high-income countries do not have a universal BCG vaccination policy, but income level and BCG coverage were independent predictors in our multivariate analysis. One possible contributing factor to the higher cases and deaths in high-income countries could be the higher median age of the population in these countries [33]. A positive correlation between the median age of the population and COVID-19 morbidity and mortality has been reported [32].

We observed minimal correlation between the CFR and the variables studies. The CFR can be affected by the lack of testing to identify cases. There is a significant difference in the testing per-capita among countries, i.e. high-income countries performing more tests per million population [34]. There was also an inverse correlation between the percentage of the population tested and the positive rate [32]. This may have caused the apparent reduction in CFR compared to the deaths per 100,000 population in high- and upper-middle-income countries. The lag-between deaths and cases may also contribute to this [8], as the pandemic is still evolving. The CFR, therefore, may be unreliable in the COVID-19 pandemic.

Non-pharmacological methods like social distancing, quarantining and isolation and changes in population behaviour have been successful in reducing rates of transmission and hospitalization [35, 36]. There is, however, no reliable worldwide data available for these measures, and we have not included them in our hypothesis.

The main limitation of this analysis is the inability to link the exposures relate to BCG vaccination to the occurrence of COVID-19 in the same person (ecological fallacy) as the data considered are at the population level. Furthermore, there may be other factors between countries that are associated with the exposure, which may account for the differences, such as comorbidities [1] and other epidemiological characteristics of the vulnerable groups, which have not been considered in the model. Several countries who do not have a universal BCG vaccination at present have stopped the vaccination in the last 20-years. Their most recent estimated BCG coverage is still high. We have also not included other variables like temperature and humidity, which have already been proven to be associated with COVID-19 [37]. The use of both BCG coverage as well as surrogate indicators of BCG coverage (i.e. incidence of pulmonary and extra-pulmonary TB, TB incidence in different age groups) has enabled a more robust evaluation of the association between BCG vaccination and COVID-19 morbidity and mortality. We did not attempt to analyse a difference between males and females, as this data was only available for a few countries.

## Conclusions

We saw a significant inverse relationship between Universal BCG vaccination policy, BCG coverage and the incidence of TB in > 65-year olds with COVID-19 cases and deaths. Although we cannot imply causality, this is a potentially useful intervention in a disease which presently has no definitive preventive or curative strategy.

## Data Availability

Available with corresponding author upon requesting.
